# Programmed cell death ligand 1 expression on monocytes is inversely correlated with tumour response to preoperative chemoradiotherapy for locally advanced rectal cancer

**DOI:** 10.1111/codi.16167

**Published:** 2022-05-24

**Authors:** Mineyuki Tojo, Hisanaga Horie, Koji Koinuma, Hideyo Miyato, Hidenori Tsukui, Yuki Kaneko, Yurie Futoh, Yuki Kimura, Kazuya Takahashi, Akira Saito, Hideyuki Ohzawa, Hironori Yamaguchi, Alan Kawarai Lefor, Naohiro Sata, Joji Kitayama

**Affiliations:** ^1^ Department of Surgery Jichi Medical University Shimotsuke Tochigi Japan; ^2^ Jichi Medical University Hospital Division of Translational Research, Center for Clinical Research Shimotsuke Tochigi Japan; ^3^ Department of Clinical Oncology Jichi Medical University Shimotsuke Tochigi Japan

**Keywords:** locally advanced rectal cancer, patrolling monocytes, preoperative chemoradiotherapy, programmed cell death ligand 1 (PD‐L1)

## Abstract

**Aim:**

The clinical efficacy of chemoradiotherapy (CRT) is largely dependent on host immune status. The aim of this study was to identify possible markers expressed on circulating mononuclear cells to predict tumour response in patients with locally advanced rectal cancer (LARC).

**Methods:**

Peripheral blood samples were obtained from 47 patients diagnosed with LARC before and after CRT. The numbers of lymphocytes and monocyte subsets were analysed using flow cytometry. Based on clinical and pathological findings, patients were classified as high or low responders.

**Results:**

Lymphocyte counts were markedly decreased after CRT. Total numbers of lymphocytes (*p* = 0.030) and CD4(+) T cells (*p* = 0.041) in post‐CRT samples were significantly lower in low responders than in high responders. In contrast, monocyte counts were not reduced and the number of CD14^dim^(+) CD16(+) nonclassical (patrolling) monocytes were somewhat increased after CRT (*p* = 0.050). Moreover, the ratios of programmed cell death ligand 1 (PD‐L1) (+) cells on patrolling monocytes before and after CRT were significantly higher in low responders than in high responders (*p* = 0.0046, *p* = 0.0006). The same trend was observed for classical and intermediate monocytes. The expression of PD‐L1 on patrolling monocytes before CRT correlated inversely with the number of T cells and natural killer (NK) cells after CRT. PD‐L1(+) ratio in patrolling monocytes was an independent predictor for response to CRT.

**Conclusion:**

Programmed cell death ligand 1 (PD‐L1) expression on patrolling monocytes suppresses cell‐mediated immunity in patients receiving CRT which could be related to tumour response, and may be a useful biomarker for decision‐making in the management of patients with LARC.

**What does this paper add to the literature?**

In 47 patients with locally advanced rectal cancer, expression levels of programmed cell death ligand 1 (PD‐L1) on monocytes in peripheral blood before chemoradiotherapy (CRT) are inversely correlated with circulating lymphocyte counts after CRT and histological tumour response. The ratio of PD‐L1(+) cells in CD16(+) patrolling monocytes is an independent predictor for response to CRT.

## INTRODUCTION

Preoperative chemoradiotherapy (CRT) is currently considered to be standard initial treatment for patients with locally advanced rectal cancer (LARC), since it can result in downstaging in approximately half of patients with locally advanced RC, resulting in a lower rate of postoperative local recurrence and a higher rate of sphincter‐preserving surgery [1, 2, 3, 4, 5]. However, the response to CRT varies greatly among patients, and CRT may have disadvantages such as delaying surgery or immune suppression in patients who do not have a response. Numerous studies have shown that many radiological findings [6, 7, 8] as well as molecular markers such as gene mutation patterns [8, 9] and protein expression patterns [9, 10] or microRNA expression [9, 11, 12] are related to the therapeutic response. However, the clinical usefulness of these biomarkers remains controversial, and none are currently in routine clinical use.

Approaches to find suitable biomarkers are based on the idea that the sensitivity to CRT is simply associated with a direct effect to induce DNA damage or apoptosis of tumour cells. However, a growing body of evidence suggests that the response of rectal tumours depends not only on direct radiocytotoxity to tumour cells, but also on the tumour microenvironment and host immune status [13, 14]. Many studies have reported that a high density of T cells at the tumour site is strongly associated with a better response to CRT [15, 16, 17, 18]. Other retrospective studies have shown that the total number of lymphocytes and their ratios to neutrophils (neutrophil lymphocyte ratio) or monocytes (lymphocyte monocyte ratio) are significantly correlated with the sensitivity to CRT and outcomes of patients with LARC [19, 20, 21, 22]. Those results strongly suggest that immunologic‐mediated cell death is certainly involved in the process of tumour regression of rectal cancer induced by CRT.

In this study, we prospectively examined the phenotypes of circulating lymphocytes and monocytes of patients with LARC before and after CRT and correlated these values with the observed histological response. We found that the expression levels of programmed cell death ligand 1 (PD‐L1) on monocyte subsets are closely related with circulating lymphocytes after CRT and the tumour response.

## METHODS

### Monoclonal antibodies and reagents

PE‐conjugated anti‐CD4 (RPA‐T4), anti‐CD16 (B73.1), APC‐conjugated anti‐CD8a (HIT8a), anti‐CD274 (PD‐L1) (29E.2A3), BV421‐conjugated anti‐CD3 (OKT3), anti‐CD14 (M5E2) and FITC‐conjugated anti‐CD279 (PD‐1) (EH12.2H7) were purchased from BioLegend Inc. FITC‐conjugated anti‐CD3 (HIT3a), BV421‐conjugated anti‐PD‐1 (EH12.1), BUV395‐conjugated anti‐CD56 (NCAM16.2) and FVS‐780 were purchased from BD Biosciences. FcR blocking reagent was purchased from Miltenyi Biotec B.V.& Co. KG.

### Patients

A total of 50 consecutive patients diagnosed with primary rectal cancer with clinical stage T2‐4 M0, were treated with a total dose of 50.4 Gy of radiation therapy and concomitant oral capecitabine (1650 mg/m^2^/day) at the Department of Surgery, Jichi Medical University between May 2017 and June 2020. Of these, 47 completed the CRT regimen without notable toxicities, and were prospectively enrolled in the study. The study protocol was approved by the Ethics Committee of Jichi Medical University (RIN‐A20‐098), and written informed consent was obtained from all patients. Most patients underwent standard total mesorectal excision, after an interval of 8–10 weeks following CRT. All resected specimens were examined pathologically, and the findings were recorded in accordance with the TNM classification. The histological regression of the primary rectal lesion in response to CRT was evaluated and classified as high or low, based on the amount of residual cancer according to the Japanese Classification of Colorectal Carcinoma, that is, lesions in which no tumour remained (grade 3) and more than two‐thirds of the cancer had degraded, necrotized or disappeared (grade 2) were classified as high responders, while those with less than two‐thirds reduction (grades 1a and 1b) were classified as low responders.

### Blood sampling and analysis of leucocyte phenotypes

Peripheral venous blood samples were obtained before neoadjuvant CRT and 8 weeks after completion of CRT, prior to surgery. Peripheral blood mononuclear cells were collected before and after CRT using mononuclear cell preparation tube‐sodium heparin (Becton Dickinson) and cell counts in the samples analysed using an automated haematology analyser (XE‐5000, Sysmex). Lymphocyte and monocyte subsets were also analysed using flow cytometry. Briefly, whole blood was treated with FACS lysing solution (Becton Dickinson) to lyse red blood cells and fixed with 1% formaldehyde. The cells (1 × 10^6^) were suspended in PBS containing 0.02% EDTA and incubated for 15 min to label dead cells with FVS‐780. After washing with PBS, the cells were incubated with 5 μl FcR blocking reagent for 10 min and immunostained with relevant mAbs for 30 min according to the manufacturer's recommendations. After washing with PBS, the cells suspension was applied to BD LSRFortessa X‐20 (Becton‐Dickinson) and antigen expression analysed using Flow Jo software (Becton‐Dickinson). In phenotype analyses, more than 10,000 events in regions gated for lymphocytes or monocytes were performed.

### Statistical analysis

Cell numbers were analysed using a Student's *t*‐test. Correlation was examined with Pearson's simple linear regression analysis. All analyses were performed with Graph Pad Prism 8 Software, and *p*‐values <0.05 were statistically significant.

## RESULTS

### Patient characteristics

Forty‐six of 47 enrolled patients underwent resection after receiving CRT. Among them, 20 and five patients had a pathological response of grades 2 and 3, respectively, categorized as high responders, while 21 patients with grades 1a or 1b were classified as low responders. One patient who had a macroscopic complete response after CRT with no detectable tumour despite multiple biopsies refused surgical resection and survived more than 2 years without recurrence, and is included among the high responders. The characteristics of the 47 patients in the high and low responder groups are summarized in Table 1. Among high responders, there were significantly more males than females. High responders tended to be more in patients with tumours confined to the rectal wall (cT < 4) on computed tomography (CT) imaging (*p* = 0.058). No other clinical factor before CRT was different between the two groups. Pathological characteristics of resected tumours were less advanced among high responders due to antitumour effects of CRT.

**TABLE 1:** Clinical and pathological features of the patients with rectal cancer who underwent preoperative chemoradiotherapy (CRT)

Variables		Low response (*n* = 21)	High response (*n* = 26)	*p*‐value
Clinical variables before CRT				
Age		61 (38–73)	63 (48–76)	0.720
Gender	(M/F)	13/8	23/3	0.043
Macroscopic appearance	(1/2/3)	0/20/1	2/22/2	0.369
Location	(Ra,Rab/Rb,RbP)	1/20	1/25	1.000
Tumour length	(cm)	7.1 (3.1–10.2)	5.8 (2.8–14.6)	0.454
Circumferential rate	(%)	70% (33%–100%)	67% (20%–100%)	0.578
Depth of invasion	(cT: ≤3/4)	14/7	24/2	0.058
Nodal metastasis	(cN: 0/1≤)	7/14	6/20	0.520
Clinical stage	(cStage: 2/3)	7/14	6/20	0.520
Histology	(tub, pap/por)	20/1	25/1	1.000
Serum CEA	(ng/ml)	4.5 (0.8–42.4)	3.7 (0.8–23.0)	0.336
Serum CA19‐9	(U/ml)	14.0 (1.0–205)	11.5 (2.0–1650)	0.822
Surgery^a^	(LAR/APR/ISR/TPE)	10/10/0/1	11/13/1/0	0.931
Pathological variables after CRT^a^				
Depth of invasion	(pt: ≤1/2≤)	0/21	9/16	0.002
Nodal metastasis	(pn: 0/1≤)	10/11	18/7	0.137
Lymphatic invasion	(Negative/positive)	10/11	23/2	0.001
Venous invasion	(Negative/positive)	3/18	19/6	<0.001
Stage	(pStage: ≤1/2≤)	1/20	15/10	<0.001

*Note:* All the patients completed neoadjuvant CRT with 50.5 Gy irradiation and oral capecitabine 1650 mg/m^2^/days. *p*‐values were evaluated with Fisher's exact test or Mann–Whitney's test.

Abbreviations: APR, abdominoperineal resection; ISR, intersphincteric resection; LAR, low anterior resection; TPE, total pelvic exenteration.

^a^ Surgery was performed in 46 patients and their pathological data are presented.

### Leucocyte subpopulations in circulating blood before and after chemoradiotherapy

**FIGURE 1:**
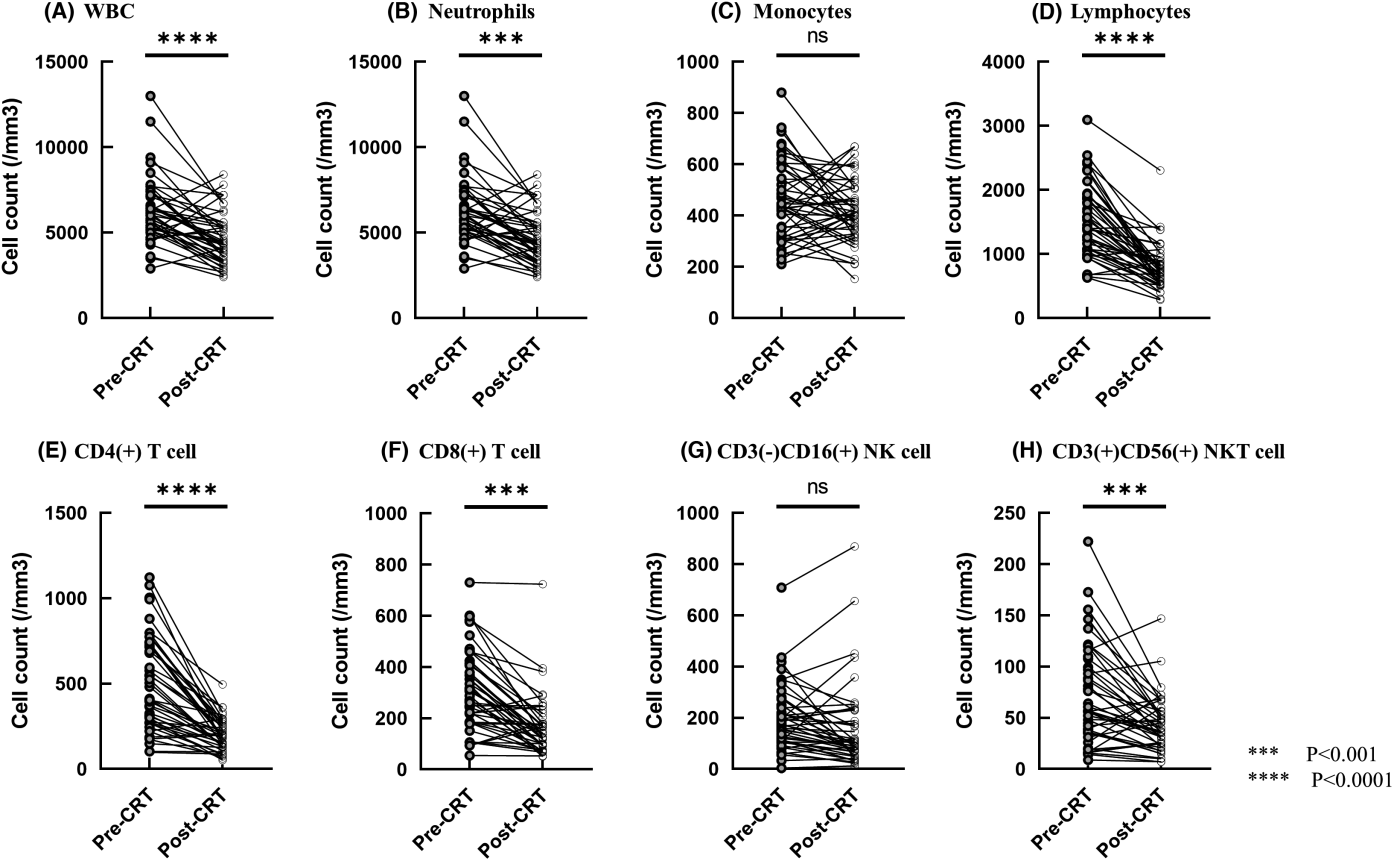
The number of peripheral white blood cells (A), neutrophils (B), monocytes (C), lymphocytes (D) and lymphocyte subsets (E–H) obtained before and after chemoradiotherapy (CRT). ***p < 0.001, ****p < 0.0001 by student *t*‐test

**FIGURE 2:**
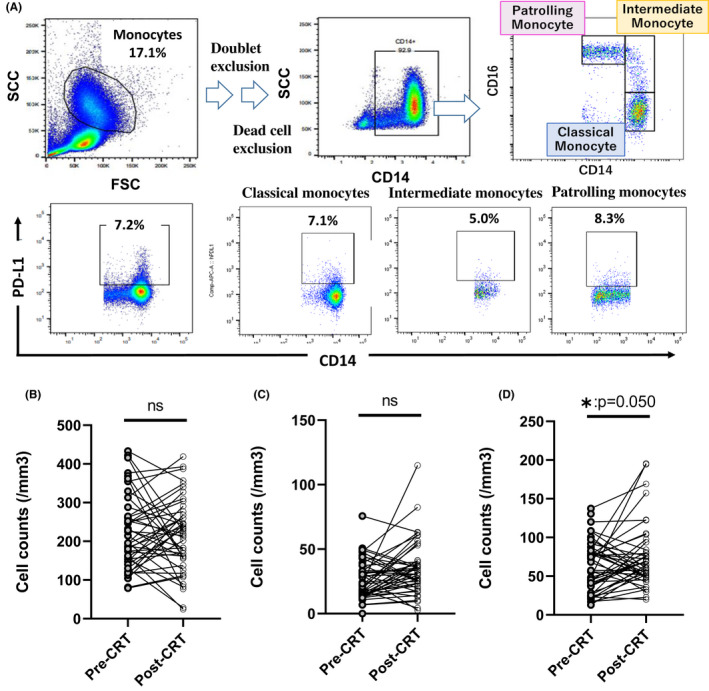
Circulating monocytes were divided into three subsets by these gating strategy (A). The number of classical (B), intermediate (C) and patrolling (D) monocytes in peripheral blood obtained before and after chemoradiotherapy (CRT). *p < 0.05 by Student's *t*‐test

The numbers of white blood cells and their subpopulations in peripheral blood were investigated from the medical records and flowcytometric analysis. As shown in Figure 1, the numbers of total leucocytes as well as neutrophils and lymphocytes were significantly reduced after CRT. The reduction rates were more prominent for lymphocytes than neutrophils (neutrophils; 4072 ± 197 to 3207 ± 197, *p* = 0.0027, lymphocytes; 1536 ± 69.2 to 791 ± 70.0, *p* < 0.0001). In contrast, the number of circulating monocytes was not significantly decreased after CRT (Figure 1C). As a result, the neutrophil‐lymphocyte and lymphocyte‐monocyte ratios were increased and decreased after CRT, respectively (Figure S1). Among the lymphocyte subpopulations, the reduction rate was most remarkable in CD4(+) T cells (511 ± 30.2 to 199 ± 30.9, *p* < 0.0001), while the number of CD3(−) CD16(+) NK cells was not changed by CRT (Figures 1E–H). As shown in Figure 2A, circulating monocytes were phenotypically divided into three subtypes, CD14^high^(+) CD16(−) classical, CD14^med^(+) CD16(+) intermediate and CD14^dim^(+)CD16(+) nonclassical (patrolling) monocytes. Although the numbers of classical and intermediate monocytes were not changed, the numbers of patrolling monocytes were increased with marginal significance (61 ± 5.4 to74 ± 5.4, *p* = 0.050).

### Leucocyte subsets and tumour response against chemoradiotherapy

The numbers of leucocyte subsets were compared with tumour response to CRT. As shown in Table 2, neutrophils, monocytes, lymphocytes and their subpopulations in blood sampled before CRT did not show any significant differences between the high and low responder groups. After CRT, however, total lymphocyte counts and the lymphocyte‐monocyte ratio in 27 patients classified as high responders were significantly higher than in low responders (685 ± 74.9 vs. 881 ± 69.2, *p* = 0.030; 1.7 ± 0.19 vs. 2.2 ± 0.18, *p* = 0.048). Among the lymphocyte subsets, the number of CD4(+) T cell was significantly higher in high responders (222 ± 18.4 vs. 173 ± 69.2, *p* = 0.041). The same trend was observed regarding CD8(+) T cells and CD3(−) CD16(+) NK cells, although the differences did not reach statistical significance. In contrast, the numbers of each monocyte subset before and after CRT were not different between the two groups.

**TABLE 2:** The number of leucocyte subsets in circulating blood before and after preoperative chemoradiotherapy (CRT) in 47 patients with locally advanced rectal cancer

Cell counts of lymphocyte or monocyte subsets (/mm^3^)	Before CRT			After CRT		
	Low response (*n* = 21)	High response (*n* = 26)	*p*‐value	Low response (*n* = 21)	High response (*n* = 26)	*p*‐value
Neutrophils	4128 ± 325	4026 ± 292	0.827	3250 ± 268	3171 ± 246	0.829
Lymphocytes	1497 ± 6125	1568 ± 113	0.678	685 ± 74.9	881 ± 68.6	0.030
CD3(+)CD4(+) T cell	519 ± 60.8	505 ± 54.7	0.868	173 ± 19.8	222 ± 18.5	0.041
CD3(+)CD8(+) T cell	313 ± 34.5	323 ± 31.1	0.833	146 ± 25.5	186 ± 23.9	0.260
CD3(+)CD56(+) NKT cell	73 ± 10.7	72 ± 9.7	0.94	46 ± 5.9	41 ± 5.5	0.467
CD3(−)CD16(+) NK cell	210 ± 25.3	176 ± 28.1	0.362	118 ± 36.2	194 ± 33.8	0.131
Monocytes	467 ± 35.5	470 ± 31.9	0.948	408 ± 26.7	423 ± 26.8	0.684
Classical monocyte	224 ± 21.7	212 ± 19.6	0.692	214 ± 20.9	215 ± 19.6	0.940
Intermediate monocyte	28 ± 3.3	26 ± 3.3	0.628	33 ± 4.6	36 ± 4.3	0.648
Patrolling monocyte	62 ± 7.3	60 ± 6.5	0.851	76 ± 9.0	72 ± 8.4	0.706
NLR	3.21 ± 0.31	2.80 ± 0.28	0.327	5.21 ± 0.50	4.10 ± 0.46	0.108
LMR	3.40 ± 0.26	3.50 ± 0.23	0.761	1.77 ± 0.19	2.11 ± 0.18	0.048

*Note:* Data show mean ± SEM, *p*‐values calculated with Student's *t*‐test. Classical, intermediate and patrolling monocytes were determined as CD14^high^(+) CD16(−), CD14^mid^(+) CD16(+) and CD14^low^(+) CD16(+) phenotype, respectively.

Abbreviations: LMR, lymphocyte monocyte ratio; NLR, neutrophil lymphocyte ratio.

### Programmed cell death ligand 1 expression on monocyte subsets and tumour response after chemoradiotherapy

**FIGURE 3:**
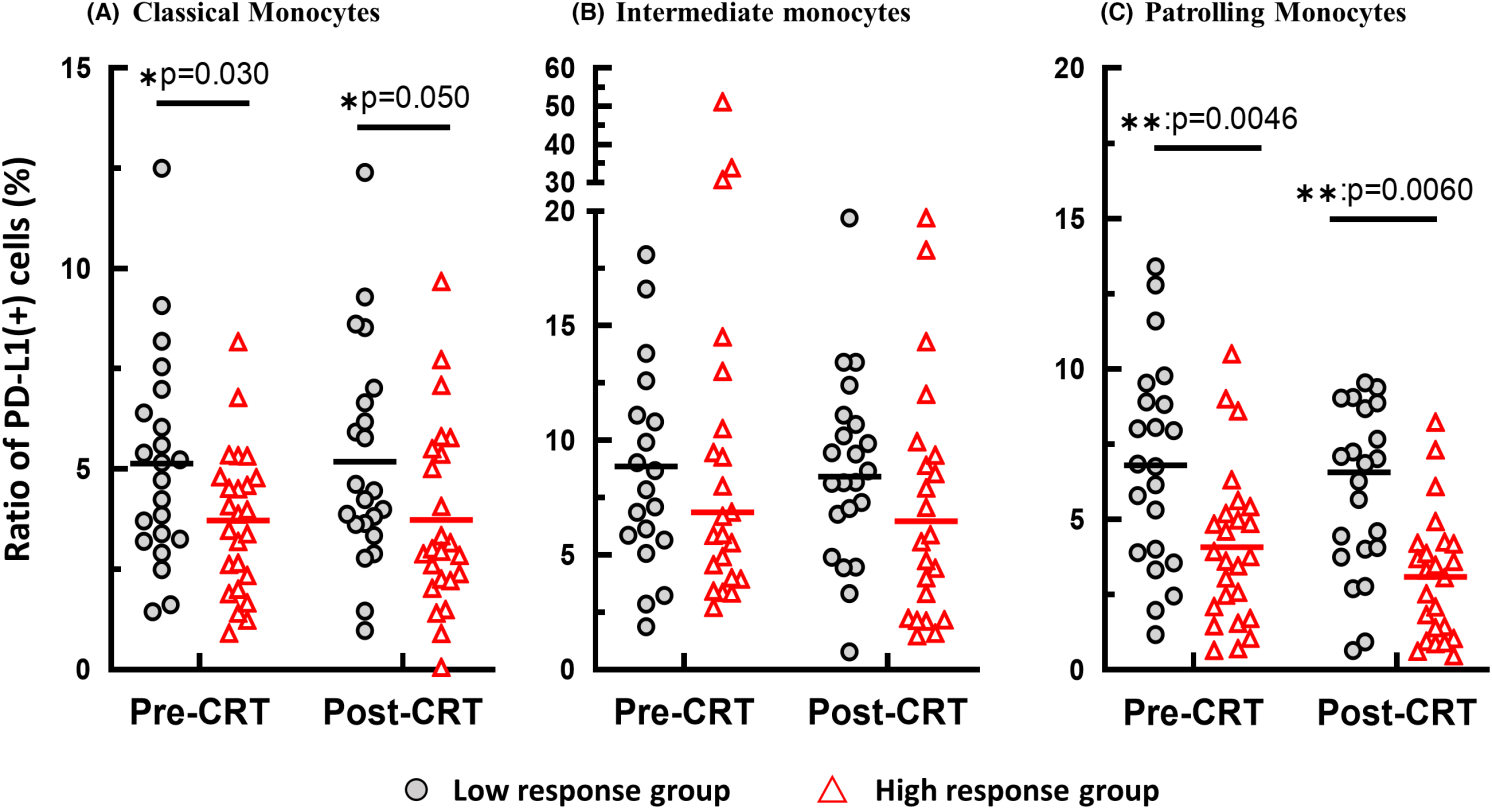
Ratio of programmed cell death ligand 1 (PD‐L1) (+) cells classical (A), intermediate (B) and patrolling (C) monocytes in peripheral blood of low and high responding patients before and after chemoradiotherapy (CRT). *p < 0.05, **p < 0.001 by student *t*‐test

We next examined the expression of PD‐L1 on each of the monocyte subsets (Figure 2A). The expression levels of PD‐L1 were not different among monocyte subpopulations which were not significantly altered by CRT. However, the PD‐L1 expression on monocytes did correlate with tumour response to CRT (Figure 3). The ratios of PD‐L1(+) cells among classical monocytes were significantly higher in the low response group than among high responders both before and after CRT (before CRT: 5.2% ± 0.48% vs. 3.7% ± 0.45%, *p* = 0.030, after CRT: 5.3% ± 0.56% vs. 3.7% ± 0.52%, *p* = 0.050; Figure 3A). The differences in PD‐L1(+) cell ratios were more pronounced in patrolling monocytes (before CRT: 6.7% ± 0.66% and 4.1% ± 0.61% *p* = 0.0046, after CRT: 5.8% ± 0.53% and 3.1% ± 0.49%, *p* = 0.0006) (Figure 3C). The same trend was shown for intermediate monocytes (Figure 3B).

### Programmed cell death ligand 1 expression on patrolling monocytes before chemoradiotherapy correlates with circulating lymphocyte counts after chemoradiotherapy

**FIGURE 4:**
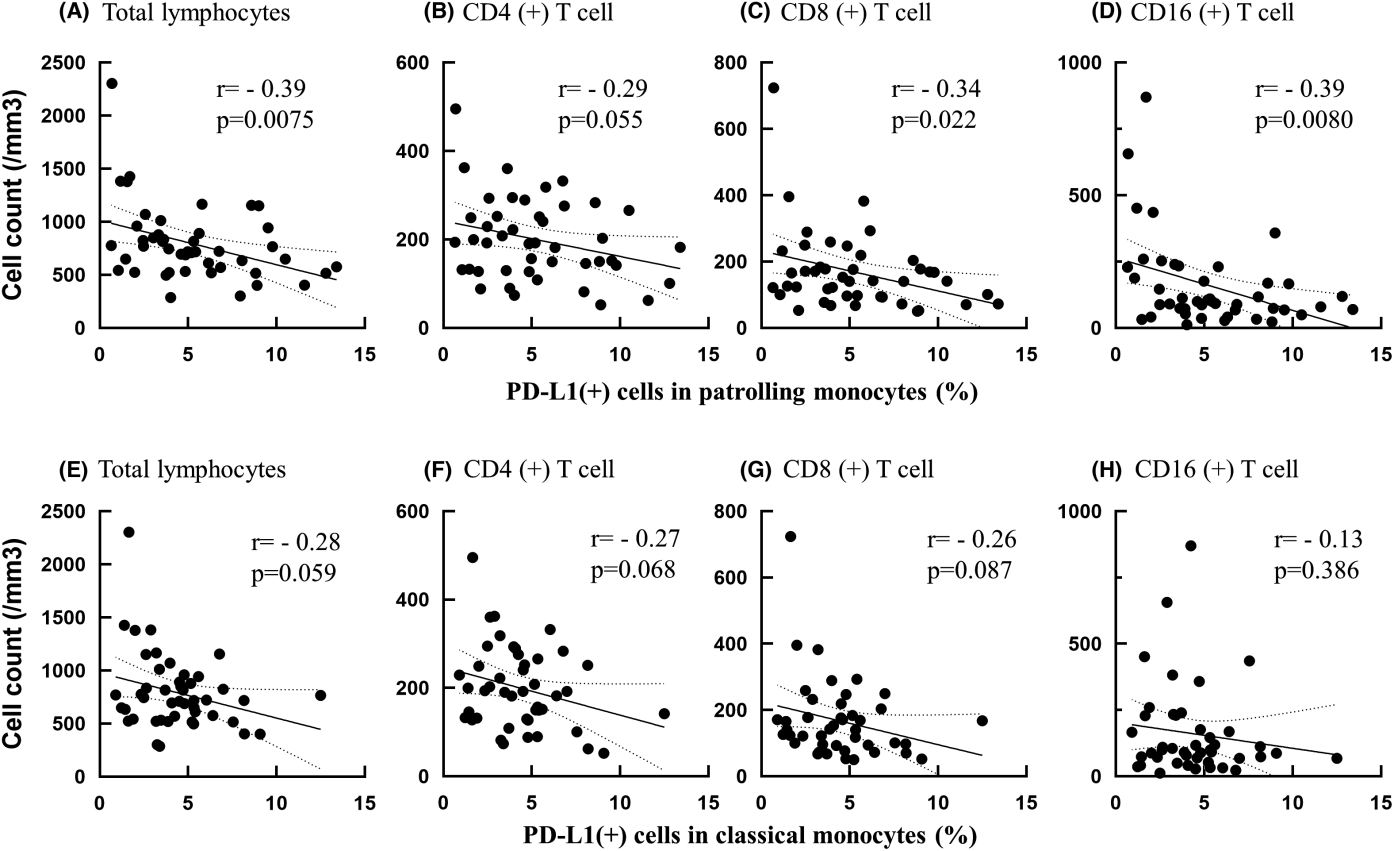
Correlation between programmed cell death ligand 1 (PD‐L1) (+) ratio in patrolling (AD) and classical (EH) monocytes in prechemoradiotherapy (CRT) blood specimens and the number of total lymphocytes and their subsets in post‐CRT specimen. Correlation efficiencies and *p*‐values were calculated with Pearson's simple linear regression analysis

As shown in Figure 4A, the ratio of PD‐L1(+) cells in patrolling monocytes before CRT showed a significant inverse correlation with lymphocyte counts in peripheral blood obtained after CRT (*r* = −0.29, *p* = 0.0075). The same correlation was observed with CD4(+) or CD8(+) T cells as well as CD3(−) CD16(+) NK cells with similar correlation coefficients (Figures 4B–D). PD‐L1 expression on classical monocytes had a similar, but weaker, correlation with post‐CRT lymphocytes counts (Figures 4E–H).

### Predictive impact of programmed cell death ligand 1 on patrolling monocytes on tumour response

**FIGURE 5:**
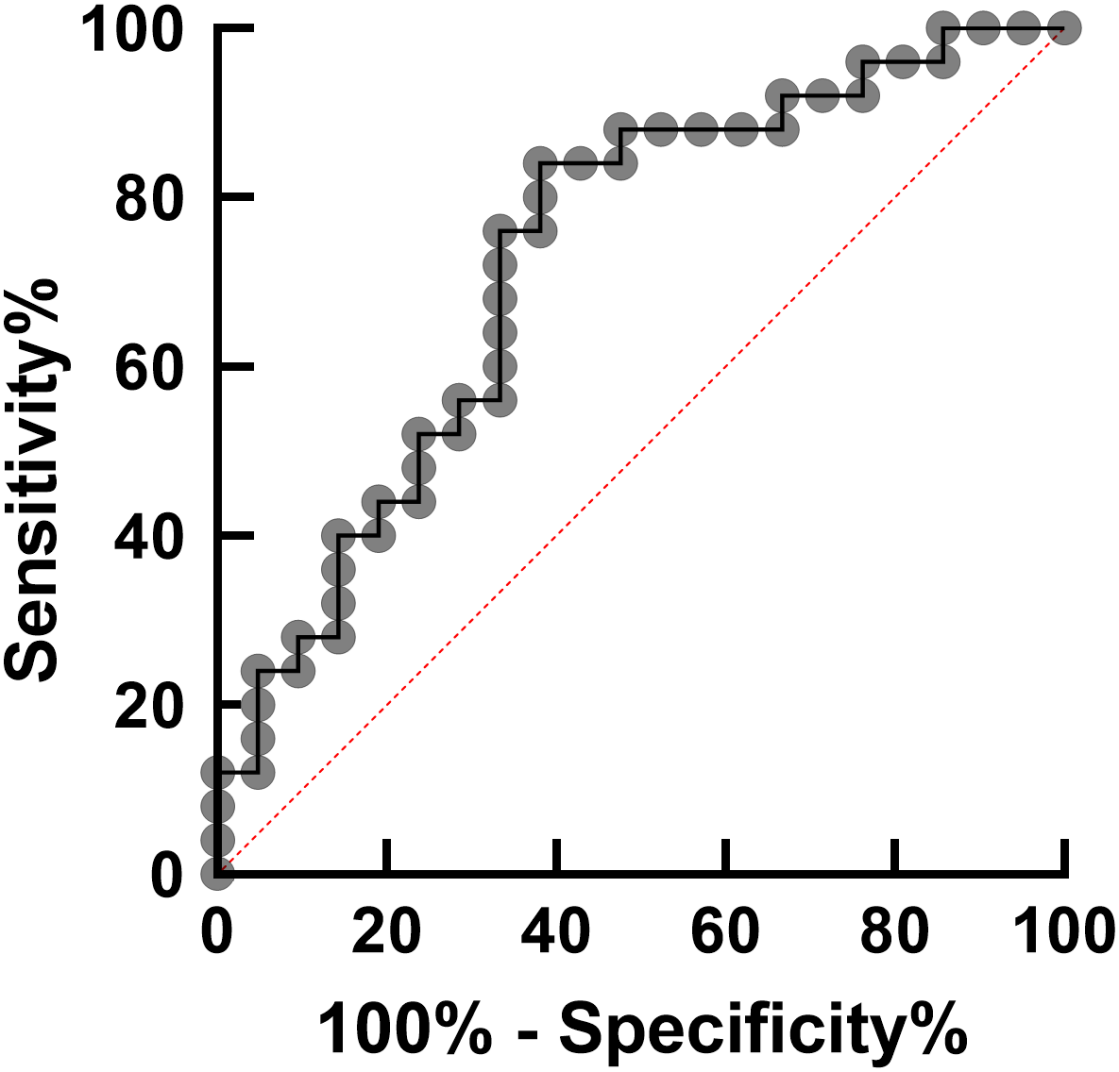
ROC curve for determining the cutoff point of programmed cell death ligand 1 (PD‐L1) (+) ratio in prechemoradiotherapy (CRT) patrolling monocytes for high response against CRT

Then, we divided the patients with the expression levels of PD‐L1 on pre‐CRT patrolling monocytes. With ROC analysis (Figure 5), the optimal cutoff value was determined as 5.2% corresponding to maximum sensitivity and specificity (76.0% and 66.7%, respectively) for predicting response against CRT. As shown in Table 3, PD‐L1(+) ratio in patrolling monocytes was selected as possible predictor for response to CRT together with gender, longitudinal tumour size, cT4, serum CEA level, neutrophil lymphocyte ratio. Multivariate analysis showed PD‐L1 (+) ratio in patrolling monocytes less than 5.2% was an independent predictor for good response to CRT.

**TABLE 3:** Univariate and multivariate analysis of clinical factors to predict high response to preoperative chemoradiotherapy (CRT) for patients with locally advanced rectal cancer

Co variables	Univariate			Multivariate		
	OR	95% CI	*p*‐values	OR	95% CI	*p*‐values
Female	0.221	0.048–0.941	0.043	0.122	0.017–0.864	0.023
Longitudinal tumour size >5.6 cm	0.313	0.094–1.040	0.080	0.367	0.070–1.924	0.227
Rab/Rb, RbP	0.275	0.028–2.671	0.362			
cT = 4	0.167	0.230–0.916	0.058	0.264	0.0429–32.375	0.213
Nodal metastasis (+)	1.667	0.460–6.034	0.520			
Circumferential tumour extent: 80%<	0.387	0.104–1.404	0.197			
Poorly differentiated histology	1.250	0.074–21.256	1.000			
CEA:10 ng/ml<	0.253	0.064–1.004	0.052			
NLR: 4.0<	0.208	0.037–1.169	0.115	0.179	0.016–1.971	0.153
LMR: <2.7	0.386	0.104–1.440	0.197	0.328	0.041–2.597	0.286
PD‐L1% in patrolling monocyte: 5.2%<	0.158	0.043–0.474	0.007	0.116	0.021–0.637	0.006

*Note:* Univariate and multivariate logistic regression analyses were performed to evaluate the impact of factors before CRT.

Abbreviations: CEA, carcinoembryonic antigen; CI, confidence interval; LMR, lymphocyte monocyte ratio; NLR, neutrophil lymphocyte ratio; OR: odds ratio.

## DISCUSSION

It was believed that both radiotherapy and chemotherapy inhibit the immune response in patients. However, previous studies have suggested that radiation therapy can induce the generation of tumour‐specific effector T cells which can promote tumour regression [23, 24] and that tumour shrinkage is largely dependent on the systemic immune response especially on T cell‐mediated cellular immunity [13, 14]. However, the number of circulating lymphocytes is generally decreased by radiation therapy and/or chemotherapy which may critically impair the anti‐tumour effects of CRT. Consistently, many retrospective studies have demonstrated that circulating lymphocyte counts correlate with tumour response in patients with LARC who received neoadjuvant CRT [19, 20, 21, 22].

In this prospective study, we confirmed that peripheral blood lymphocyte counts obtained after CRT are markedly reduced and a decreased lymphocyte count, especially CD4(+) T cells, was associated with a poor histological response. This is consistent with the results of previous studies [19, 25, 26], suggesting that CD4(+) T cells in circulating blood may play a central role to evoke an immune response against tumour‐specific antigens induced by CRT.

In contrast, the number of circulating monocytes and their subsets were not decreased after CRT. Monocytes are phenotypically divided into classical, intermediate and nonclassical (patrolling) monocytes with different functions [27]. CD14^high^(+) CD16(−) classical monocytes are the primary cells recruited in inflammatory tissue, differentiate into macrophages and play critical roles to regulate the local immune response. CD14^dim^(+) CD16(+) nonclassical monocytes migrate in vascular beds to search for harmful micro particles or damaged endothelial cells and promote their removal to maintain vascular homeostasis and are referred to as patrolling monocytes [28]. Although patrolling monocytes are generally considered to be the first line of defence for the recognition and clearance of pathogens, their roles in tumour biology remain unclear. Recent preclinical studies have suggested that patrolling monocytes can prevent tumour metastases through the activation of NK cells [29, 30, 31]. In contrast, Jung et al. showed that Ly6C^lo^ patrolling monocytes can exert immunosuppressive roles to reduce the effects of anti‐VEGFR2 therapy [32]. These results suggest that patrolling monocytes may play divergent roles in different phases of tumour progression.

In this study, it was shown that the number of patrolling monocytes, but not other types of monocytes, increased after CRT. However, the number of these monocytes did not significantly correlate with tumour response. We then examined the expression of PD‐L1 on these monocyte subsets, since the PD‐1/PD‐L1 axis is believed to play a critical role in suppressing the immune system and mediating evasion of host immune surveillance in malignant tumours [33]. Interestingly, expression of PD‐L1 on circulating monocytes had a strong inverse correlation with tumour response to CRT. The ratio of PD‐L1 (+) cells among classical and patrolling monocytes were significantly higher in patients with low response group than counterparts. The difference was more prominent in patrolling monocytes, and the same trend was observed regardless of the timing of blood sampling.

Programmed cell death ligand 1 is known to be expressed not only on tumour cells but also on stromal and haematopoietic cells and recent preclinical studies have suggested that expression of PD‐L1 on host myeloid cells has a stronger impact on antitumour immunity than PD‐L1 present on tumour cells [34, 35, 36]. Previous studies have shown that the level of PD‐L1 expression on circulating monocytes is strongly related to the progression of infectious diseases [37, 38, 39]. More recently, de Coana et al. [40] have shown that high expression of PD‐L1 on monocyte subpopulations correlates with shorter survival in patients with melanoma who received PD‐1 blockade therapy, suggesting that expression of PD‐L1 on monocytes may affect tumour response to cytotoxic therapies.

The roles of PD‐L1 expression on monocytes in tumour immunity are largely unknown. This is the first report to show a correlation between PD‐L1 expression on circulating monocytes and tumour response to CRT. In this study, the ratio of PD‐L1(+) cells among monocytes, especially patrolling monocytes, showed an inverse correlation with the number of circulating T cells and NK cells after CRT. Moreover, PD‐L1 on patrolling monocytes was an independent predictor for response to CRT with multivariate analysis. These facts suggest the possibility that PD‐L1 expression on monocytes has a suppressive effect on lymphoid haematogenesis during CRT, which may result in impaired cell‐mediated immunity to reduce the tumour response. Recently, multiple clinical trials have suggested synergistic effects between radiation therapy and immunotherapy using immune checkpoint inhibitors [41, 42, 43]. However, such benefits have not been confirmed in other clinical studies [44, 45] and the mechanisms of the synergism remain unclear. The results of this study suggest that PD‐L1 expression on monocytes may affect the response to combination therapy and may be useful as a biomarker to determine the optimal neoadjuvant treatment for patients with LARC.

## CONFLICT OF INTEREST

All authors declare that there are no conflict of interests.

## Supporting information


Figure. S1

## Data Availability

The data that support the findings of this study are available from the corresponding author upon reasonable request.
