# Supplementary figures and images for: Programmed cell death ligand 1 expression on monocytes is inversely correlated with tumour response to preoperative chemoradiotherapy for locally advanced rectal cancer
Source: Colorectal Dis. 2022 May 24;24(10):1140–9. doi: 10.1111/codi.16167 (PMC9790410; doi:10.1111/codi.16167)

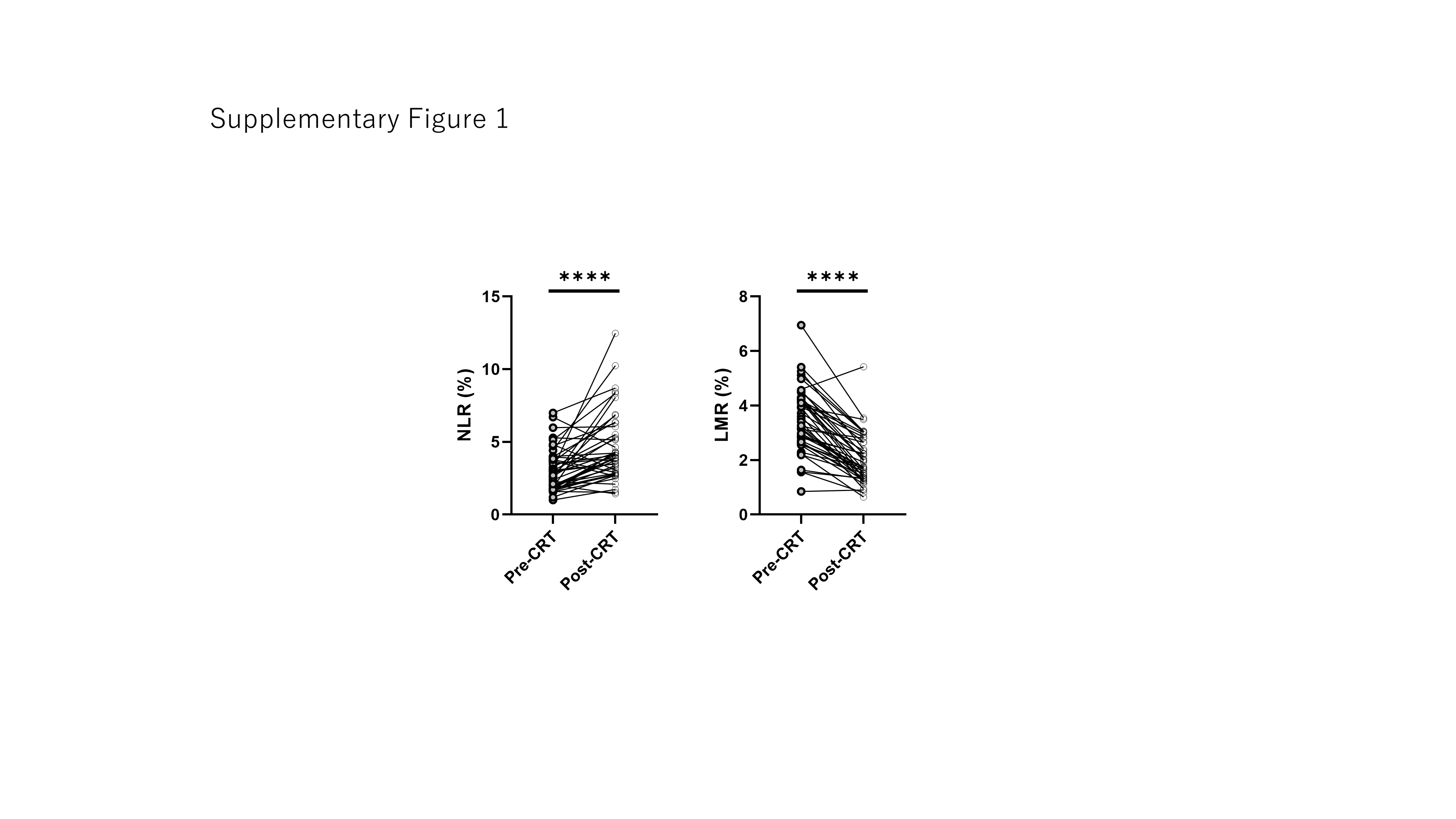

Supplement: Supplementary file 1 — Figure. S1 [file CODI-24-1140-s001.tif]
